# PLK4 initiates crosstalk between cell cycle, cell proliferation and macrophages infiltration in gliomas

**DOI:** 10.3389/fonc.2022.1055371

**Published:** 2022-12-22

**Authors:** Xiaoyang Zhang, Zesheng Li, Cheng Wei, Lin Luo, Shenghui Li, Junhu Zhou, Hao Liang, Ying Li, Lei Han

**Affiliations:** ^1^ Tianjin Neurological Institute, Key Laboratory of Post-Neuroinjury Neuro-repair and Regeneration in Central Nervous System, Ministry of Education and Tianjin City, Tianjin Medical University General Hospital, Tianjin, China; ^2^ Department of Neurosurgery, The First Affiliated Hospital of Zhengzhou University, Zhengzhou, Henan, China

**Keywords:** PLK4, glioma, tumor immune microenvironment, macrophage, cancer

## Abstract

Tumor immune microenvironment plays an important role in tumorigenesis and metastasis. Polo-like kinases 4 (PLK4) is a crucial regulatory factor in the process of cell cycle, and its abnormal regulation often leads to a variety of diseases including tumorigenesis. We have previously explored the function of PLK4 in sensitizing chemotherapy in glioma, but there are few studies on the correlation between PLK4 and tumor immune microenvironment. PLK4 was found to be highly expressed in various types of cancers, including glioma and closely related to histological and genetic features in public databases. Kaplan-Meier survival analysis and Cox regression analysis revealed that higher PLK4 expression is associated with poorer prognosis. GO and KEGG functional enrichment analysis showed that PLK4 expression level was significantly correlated with regulation of immune microenvironment, cell cycle and genomic instability. Immune infiltration analysis showed that high expression of PLK4 resulted in reduced infiltration of macrophages. M1 macrophage infiltration assays showed that PLK4 knockdown GBM cell lines promoted the recruitment of M1-type macrophages *via* altering expression of chemokines. And in intracranial tumor mouse models, PLK4 inhibition increased tumor-infiltrating M1 macrophages. In summary, our results demonstrated the correlation between high PLK4 expression level and malignant progression of gliomas, and the possible involvement of PLK4 in regulation of cell cycle, cell proliferation and macrophages infiltration in gliomas.

## Introduction

Because of the complexity of tumorigenesis and tumor development, the functional analysis of genes across various cancer types is essential for estimating clinical prognosis and studying the molecular mechanisms underlying carcinogenic factors. As core members of serine/threonine kinase family, polo-like kinase (PLK) is a double-edged sword, exerting multiple effects in both beneficial and adverse aspects. PLKs play crucial roles in balancing cell cycle, ensuring the maintenance of cell survival. On the other hand, PLKs might serve as potential factors that potentiate tumorigenesis and progression. So, it is necessary to further explore its function. PLKs are widespread in eukaryotic cells. The human PLKs family includes PLK1; PLK2; PLK3; PLK4 and PLK5. Polo-like kinase 4 (PLK4) functions as a serine/threonine-protein kinase attribute to the redundant catalytic domain, named the Polo-box 3 (PB3) domain (1). PLK4 regulates the cell cycle and centrosome duplication; thus, PLK4 is critical for cell mitosis and DNA damage responses (2, 3). In addition, abnormal PLK4 expression leads to abnormal centrosome numbers, promoting genomic instability and tumorigenesis (3). Previous studies have elucidated a variety of additional functions for PLK4 in cancers. For example, PLK4 enhances the invasion and metastasis capabilities of breast cancer cells by regulating the actin cytoskeleton *via* the actin-related protein (ARP) 2/3 complex (4). A previous study by our group demonstrated that PLK4 could enhance the sensitivity to chemotherapy in gliomas by phosphorylating the inhibitor of nuclear factor-kappa B kinase subunit epsilon (IKBKE) (5). However, although many databases—such as the Cancer Genome Atlas (TCGA), the Gene Expression Omnibus (GEO), and the Genome-Tissue Expression (GTEx) database—exist, few studies have examined the pan-cancer carcinogenic effects of PLK4.

In this study, we comprehensively characterized the PLK4 expression and its correlation with the prognosis of tumor patients in pan-cancer. Given our expertise in neurosurgery, we focused additional analyses on glioma. TCGA and CGGA datasets were employed for further validation of the correlation between PLK4 mRNA expression and glioma grades or prognosis. Besides, the hypothesis that PLK4 is closely related to tumor immune microenvironment, cell cycle progression, and genome instability was also supported by Gene Ontology (GO) and Kyoto Encyclopedia of Genes and Genomes (KEGG) enrichment analysis of its related genes in pan-cancer, especially in glioma. *In vitro* experiments such as flow cytometry, CCK8 and EdU also confirmed that aberrant expression of PLK4 potentiates glioma by disordering the cell cycle. We then further investigated the relationship between PLK4 and immune cell infiltration, showing that there is a crosstalk network in the GBM microenvironment involving the regulation of cell cycle, cell proliferation and macrophages infiltration. The CFI-400945 (PLK4 inhibitor) was administered to mice bearing intracranial murine tumor, and the efficacy was significantly proved by living imaging and HE staining. Immunofluorescence staining further revealed the relationship between overexpressed PLK4 and decreased M1 macrophage infiltration.

## Materials and methods

### mRNA expression analysis

We used the “Gene” module of SangerBox (6), a web-based program (http://www.sangerbox.com), to examine the mRNA expression levels of PLK4 in different tumor and normal tissues as reported by TCGA and performed an integrated analysis of the TCGA (7) and GTEx (8) databases. Furthermore, several TCGA tumors were analyzed using the “Pathological Stage Plot” module of GEPIA2. Meanwhile, a Chinese Glioma Genome Atlas (CGGA) download of PLK4 mRNA expression data of glioma was also done (9) (http://www.cgga.org.cn; dataset ID: mRNAseq_325, mRNAseq_693, and mRNA-array_301). The row data was merged and normalized. A correlation between PLK4 expression levels and tumor grade was then analysed using GraphPad Prism 8.0 software using clinical data from glioma patients. Specifically, we obtained patients’ follow-up time and PLK4 gene expression. Roc analyses were performed at 365, 1095, and 1825 time points using pROC ROC functions, and AUC and confidence intervals were evaluated using pROC CI functions to obtain the final AUC results. PLK4 mRNA expression data of glioma with different WHO gardes (WHO II, III, and IV), three new types (oligodendroglioma, astrocytoma and glioblastoma), molecular subtypes (IDH status, 1p/19q codeletion status and MGMT promoter status), and four subtypes (classical, mesenchymal, neural, and proneural) were obtained from the TCGA, CGGA_325, and CGGA_693 databases. To compare scores between groups, a one-way ANOVA was used. We considered *P*-values less than 0.05 to be statistically significant.

### Protein expression analysis

We applied the “HomoloGene” function (https://www.ncbi.nlm.nih.gov/homologene/) of the NCBI (10) (National Center for Biotechnology Information) to conduct conserved functional domain to analyze the PLK4 protein in different species. We obtained a phylogenetic tree of PLK4 protein sequences in different species using the constraint-based, multiple alignment online tool provided by NCBI (https://www.ncbi.nlm.nih.gov/tools/cobalt/). Using the Human Protein Atlas (HPA) database (11) (https://www.proteinatlas.org/humanproteome/pathology), we obtained protein location and gene expression data of the PLK4. We used the “Tissue Atlas” module to analyze differences in PLK4 protein expression levels among normal tissues and the “Pathology Atlas” module to analyze differences in PLK4 protein expression in different tumors *via* immunohistochemical (IHC) staining using two primary antibodies (HPA035026, HPA043198).

### Survival prognosis analysis

From the TCGA database, GEPIA2 online website provided data on the overall survival (OS) and disease-free survival (DFS) survival maps for PLK4. With the cutoff-high (50%) and cutoff-low (50%) expression thresholds, the high-expression and low-expression cohorts of PLK4 were obtained. A log-rank *P*-value survival plot was analyzed with GEPIA2’s “Survival Analysis” module. On the SangerBox portal, data for COX_OS, COX_DFS, COX_DFI (disease free interval), and COX_PFI (progression free interval) analysis of PLK4 is analyzed for different tumor types. *P*-values were calculated using log-rank data, followed by hazard ratio (HR) and 95% confidence intervals. We analyzed PLK4 expression in glioma IDH-mut+1p/19q codeletion and non-codeletion groups, as well as IDH wild groups through these three databases. In addition, the expression of PLK4 in glioma in TCGA, CGGA_693, and CGGA_325 datasets were analyzed for its impact on prognosis. Data from CGGA_325, CGGA_693, and TCGA datasets was analyzed with Cox regression using SPSS and visualized with SangerBox using nomogram.

### PLK4-related gene enrichment analysis

In the protein name module of the STRING website (https://string-db.org/), “PLK4” was queried (12). In the organism module, “Homo sapiens” was queried. In addition to the definition of network edges (evidence), the minimum interaction score of low confidence (0.150) was established, active interaction sources were determined (experiments and database), and the maximum number of interactors to show was set (no more than 50 interactors in the first shell). PLK4 and the 100 PLK4-associated targeting genes were compared using GEPIA2’s “correlation analysis” module. The *P*-value and the correlation coefficient (R) were generated. In order to generate the correlation scatter plot, we used the log2 (TPM) method for 10 genes. These 100 PLK4-related genes were analyzed using GeneDenovo for Gene Ontology (GO) and Kyoto Encyclopedia of Genes and Genomes (KEGG) pathway analyses.

### Immune-related analysis

A study was carried out using the SangerBox website to examine the relationship between PLK4 expression and ESTIMATE Score, or tumor infiltration immune cells (TIICs) in multiple tumor types. We studied the relationship between PLK4 expression and immune cells infiltration of GBM in CGGA_325, CGGA_693, and TCGA datasets using ImmunCellAI (13) portals. Using the R package GSVA, gene set variation analysis (GSVA) was performed on metagenes. To evaluate immune cell infiltration in TCGA datasetsand, the quanTIseq algorithms were used by Spearman’s rank correlation test.

### Genetic alternations

The Tumor Immune Estimation Resource 2.0 (TIMER2) website was used to investigate the genetic mutation ratios of PLK4 in various tumors (http://timer.cistrome.org/) (14). The online cBioPortal database (https://www.cbioportal.org/) (15) was then used to explore the characteristics of genetic alterations in PLK4. From TCGA datasets, somatic mutation databases and somatic copy number alternations (CNAs) were compiled. The threshold copy number at alteration peaks and the correlation of CNAs with PLK4 expression have been analyzed using GISTIC 2.0 (https://cloud.genepattern.org/) (16). According to the expression value of PLK4 in the patients, 25% were classified as PLK4^high^ and 25% as PLK4^low^. As well, the somatic mutations of patients with 25% PLK4^high^ glioma and 25% PLK4^low^ glioma were visualized in R by using the maftools package (17).

### Single-cell RNA analysis

The single-cell data expression matrix (GSE84465) was processed with the R package Seurat. We employed “NormalizeData” to normalize the gene expression data, followed by utilizing “FindVariableGenes” to identify 2,000 highly variable genes (HVGs). Then, we performed principal component analysis (PCA) with “RunPCA”. “FindNeighbors” and “FindClusters” function were employed to further cluster cells. Visualization was performed using “TSNE” and annotation was done with “SingleR” R package. PLK4 expression was visualized using “VlnPlot”. “Monocle” packages were used to reconstruct pseudotime trajectory for single cells.

### Cell culture

The human astrocyte was grown in Astrocyte Medium (AM) (ScienCell, USA) medium supplemented with 10% fetal bovine serum (ThermoFisher Scientific, USA). Human glioblastoma cell lines (U87, U251, LN229, A172 and T98) and murine glioblastoma GL261 cells were cultivated in DMEM medium supplemented with 10% fetal bovine serum (ThermoFisher Scientific, USA). THP-1 monocytes are maintained in culture in RPMI-1640 medium with 10% fetal bovine serum (Thermo Fisher Scientific, USA). All cells have been cultivated at 37°C in a humidified atmosphere (95% humidity) with 5% CO_2_.

### SiRNA transfection

PLK4 expression was knocked down in U87 and LN229 cell lines by siRNA (GenePharma, Shanghai, China). In knockdown experiment, GBM cells (U87MG and LN229) were treated with PLK4-siRNA by using Lipofectamine RNAiMAX (ThermoFisher Scientific, USA). The cells were collected at 48 h for RNA expression detection. Two PLK4 siRNAs were designed to avoid the off-target effect, PLK4 siRNA#1 sense, 5’-ACUCCUUUCAGACAUAUAAGTT-3’; antisense, 5’-CUUAUAUGUCUGAAAGGAGUTT-3’; PLK4 siRNA#2 sense, 5’-CUAUCUUGGAGCUUUA UAATT-3’; antisense, 5’-UUAUAAAGCUCCAAGAUAGTT-3’. The meaningless sequence, si-NC: sense 5’-UUCUUCGAACGUGUCACGUTT-3’; antisense, 5’-ACGUGACACGUUCGGAGAATT-3’.

### Quantitative real-time PCR

The expression of PLK4 mRNA were detected by qRT-PCR. An extraction of total RNA was performed using TRIzol (Invitrogen, USA). We reverse-transcribed mRNA (1µg) into cDNA using a kit for reverse transcription (Takara, Japan). We employed the GoTaq^®^ qPCR Master Mix with the ABI QuantStudio 3 for measuring mRNA expression (Promega, USA). mRNA expression analysis used GAPDH as a loading control. Amplification of cDNA using GoTaq^®^ qPCR Master Mix and each primer was performed. All reactions were performed in duplicate. The primer sequences were shown in **Table 1**. Data were analyzed using the relative standard curve method and normalized to GAPDH. The statistical analysis was conducted using a *t* test, with a significance level of *P*<0.05.

**Table 1:** Primers sequence used in the article.

Gene	Forward	Reverse
PLK4	5’-GACACCTCAGACTGAAACCGTAC-3’	5’-GTCCTTCTGCAAATCTTGGC-3’
GAPDH	5’-GGTGGTCTCCTCTGACTTCAACA-3’	5’-GTTGCTGTAGCCAAATTCGTTGT-3’
CXCL1	5’-CTGGCGGATCCA AGCAAATG-3’	5’-GCCCCTTTGTTCTAAGCCAG-3’
CXCL2	5’-AGCTTGTCTCAACCCCGCATC-3’	5’-CCTTCAGGAACAGCCACCAATA-3’
CXCL3	5’-CGCCCAAACCGAAGTCATAG-3’	5’- GCTCCCCTTGTTCAGTATCTTTT-3’
CXCL5	5’- CAGACCACGCAAGGAGTTCA-3’	5’-CTTCCACC TTGGAGCACTGT-3’
CXCL6	5’- ACGCTGAGAGTAAACCCCAA-3’	5’- CCAGACAAACTTGCTTCCCG-3’
CXCL7	5’-CTTGGCGAAAGGCAAAGAGG-3’	5’-GCAATGGGTTCCTTTCCCGA-3’
CXCL8	5’-TTCAGAGACAGCA GAGCACAC-3’	5’- ACTCCTTGGCAAAACTGCAC-3’
CCL2	5’- CCCAAAGAAGCTGTGATCTTCA-3’	5’-TCTGGGGAAAGCTAGGGGAA-3’
CCL3	5’- AGCCCGGTGTCATCTTCCT-3’	5’- ATGTTCCCAAGGCTCAGGC-3’
CCL5	5’- TTGCCTGTTTCTGCTTGCTC-3’	5’- AACTGCTGCTGTGTGGTA GAA-3’
CCL7	5’-TTGCTCAGCCAGTTGGGATTA-3’	5’-TGGCTACTGGTGG TCCTTCT-3’
CCL8	5’-TGCTGAAGCTC ACACCCTTG-3’	5’- TGGAATGGAAACTGAATCTGGC-3’
CCL23	5’- ACTTCTGGACAGATTCCATGCT-3’	5’-GAGCACTCGCTGTTCGTTTC-3’

### Western blot assay

A cocktail of protease and phosphatase inhibitors (Sigma, USA) was added to RIPA buffer (Pierce, France) to lyse cells. BCA assay kit was used to measure protein concentrations (Solarbio, China). After SDS-PAGE, 20 ug of protein lysates were transferred to PVDF membranes (Millipore, USA). As the primary antibodies were incubated overnight at 4°C with the secondary antibody, the corresponding HRP-conjugated secondary antibodies were incubated with the membranes. PLK4 (1:250 dilution, Abcam, ab2642) and GAPDH (1:1000 dilution, Cell Signaling Technology, 97166) were incubated at the same time. The loading control was GAPDH. The statistical analysis was conducted using a *t* test, with a significance level of *P* > 0.05.

### EdU assay

To confirm the effect of PLK4 on the proliferative activity of glioma cells, EdU kit (Cell-Light EdU Apollo567) was purchased from Guangzhou Ruibo Biotechnology Co., LTD. Four thousand cells were planted in each well of 96-well plate, and then PLK4 siRNA was transfected. EdU was stained according to the kit instructions at the set time point, and images were collected by fluorescence microscope (IX81, Olympus Company, Japan) for analysis, and the positive rate of EdU was calculated. That is, the number of EdU positive cells in the three fields randomly divided by the number of nuclei in the field. A *t* test was used for statistical analyses, and *P* < 0.05 was considered statistically significant.

### Induced M1-like macrophages and M1-like macrophages infiltration assay

Phorbol 12-myristate 13-acetate (PMA; P6741, Solarbio, China) was used to differentiate THP-1 cells into macrophages. Macrophages were polarized to M1 macrophages by incubation with IFN-γ (30002, PeproTech, China) and LPS (L8880, Solarbio, China) for 24 h. M1 macrophages without serum were seeded in the upper chamber (Corning, NY, USA). The U87 cells (1.0×10^5^) were cultured with 10% FBS in DMEM at the bottom plate. After 48 h incubation, the migrated M1 macrophages were counted in the bottom chamber. Each experiment was performed three times.

### CCK8 assay

Cell Counting Kit-8 (Dojindo, Japan) was employed to examine the cell viability. Approximately two thousand cells were planted in 96-well plates and then PLK4 siRNA was transfected. The days for measurement were set at 0, 1, 2, 3, 4 and 5 days after the cells were transfected. The cells were treated with 10 μl CCK-8. One hour later, the OD values at 450 nm were measured. The *t* test was applied for statistical analyses, and *P* < 0.05 was considered statistically significant.

### Animals

Female C57BL/6 wild-type mice (4 weeks old, 16-20g) were used in our study. Four animals were housed per cage. The mice used in our study were supplied by the Beijing HFK Bio-Technology (Beijing, China). All the experimental procedures were approved by the Animal Ethical and Welfare Committee of Tianjin Medical University.

### Mouse model of glioma

For establish the intracranial tumor model, GL261 cells were infected with luciferase lentivirus (Genechem, China). Ketamine and xylazine (75 and 7.5 mg/kg, respectively) were injected to anesthetize the C57BL/6 mice. Then, the cells were injected with a 10-μl Hamilton microsyringe. The infusion pump was utilized to control the infusion rate at 1 μl/min × 2 min (in a total of 2 × 10^5^ cells per mouse). The injection site was selected at a depth of 3.0 mm in the right striatum of C57BL/6 wild-type mice (coordinates of bregma: 2.0 mm laterally). Standardized operations were adopted throughout the surgical procedures to avoid technical differences. The mice were equally divided into 2 experimental groups: C57BL/6 with CFI400945 administration; C57BL/6 with vehicle administration. CFI400945 (7.5 mg/kg) and the vehicle were administered once daily for 21 days by gavage. Bioluminescent imaging of tumor growth was performed on days 7, 14, 21 and 28 of anesthesia in live mice by injecting D-luciferin intraperitoneally (15 mg/kg, beetle luciferin potassium salt, E1605, Promega) 15 minutes before imaging using an IVIS imaging system (Perkinelmer, USA) for 10-120 seconds. Three weeks after implantation, three animals per group were sacrificed and brain samples were collected for immunofluorescence staining and HE staining. Bioluminescent imaging was performed on the mice remaining in each group.

### HE and immunofluorescence staining

Tumor tissues were fixed in 10% neutral buffered formalin for hematoxylin and eosin (HE) staining and immunofluorescence analysis. For the experimental procedure, please refer to a previous article published of our research group (5).

### Statistical analysis

SPSS 16.0 and GraphPad Prism 8.0 were employed for mapping and statistical analysis. All RNA sequencing data were standardized. In order to compare the two groups, we used an independent sample *t* test to compare continuous variables with normal distributions. Log-rank tests were used to compare the survival differences between low expression and high expression groups after drawing a Kaplan-Meier survival curve. To determine the significance of survival differences, HRs and *P*-values were used. An assessment of gene expression associations was conducted using Pearson’s correlation coefficients and statistical significance, and the correlation’s strength was determined by its absolute value. The results were regarded as statistically significant at *P < 0.05, **P < 0.01 and ***P < 0.001.

## Results

### PLK4 mRNA expression analysis data

**Figure 1:**
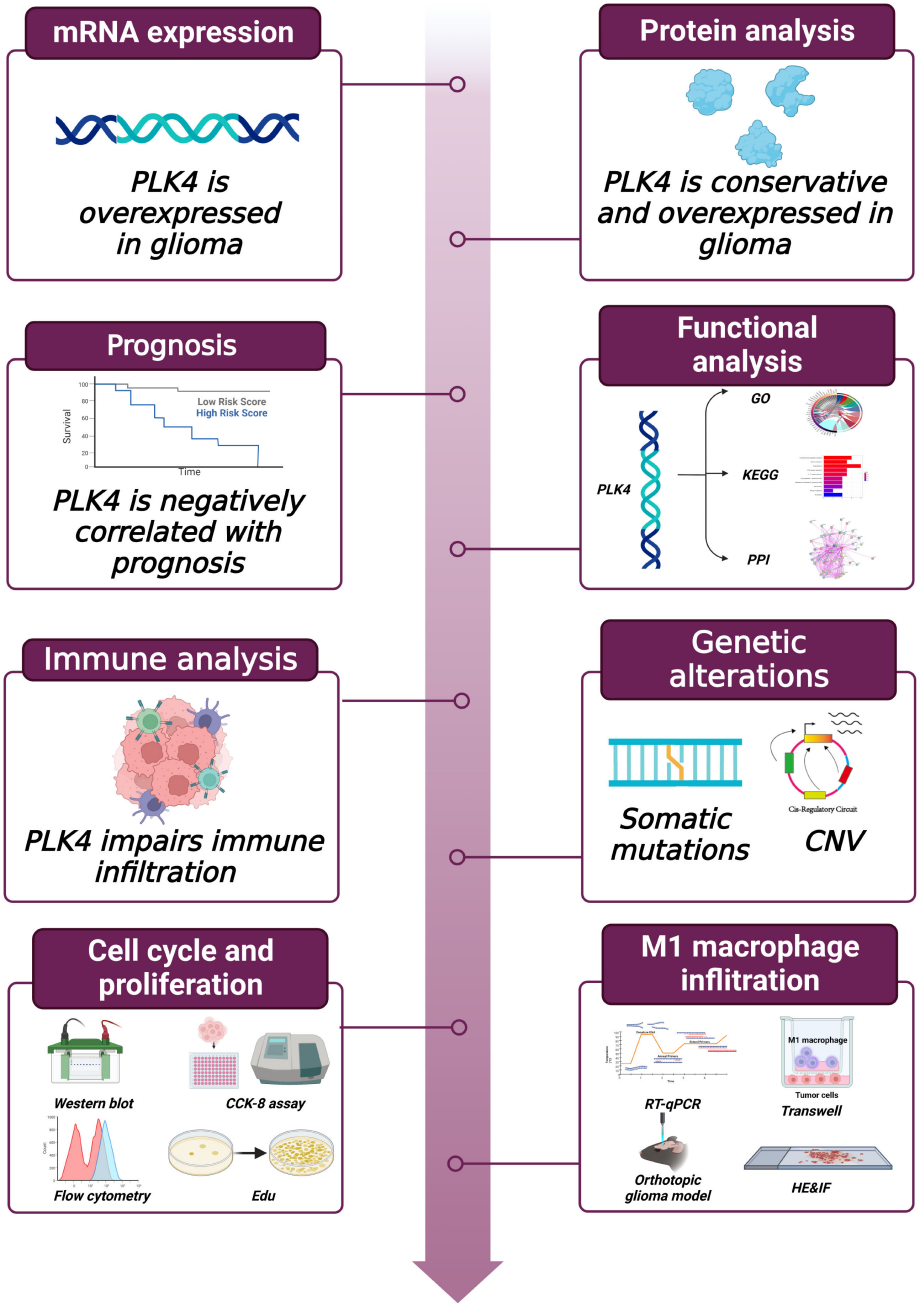
Workflow of this study.

**Figure 2:**
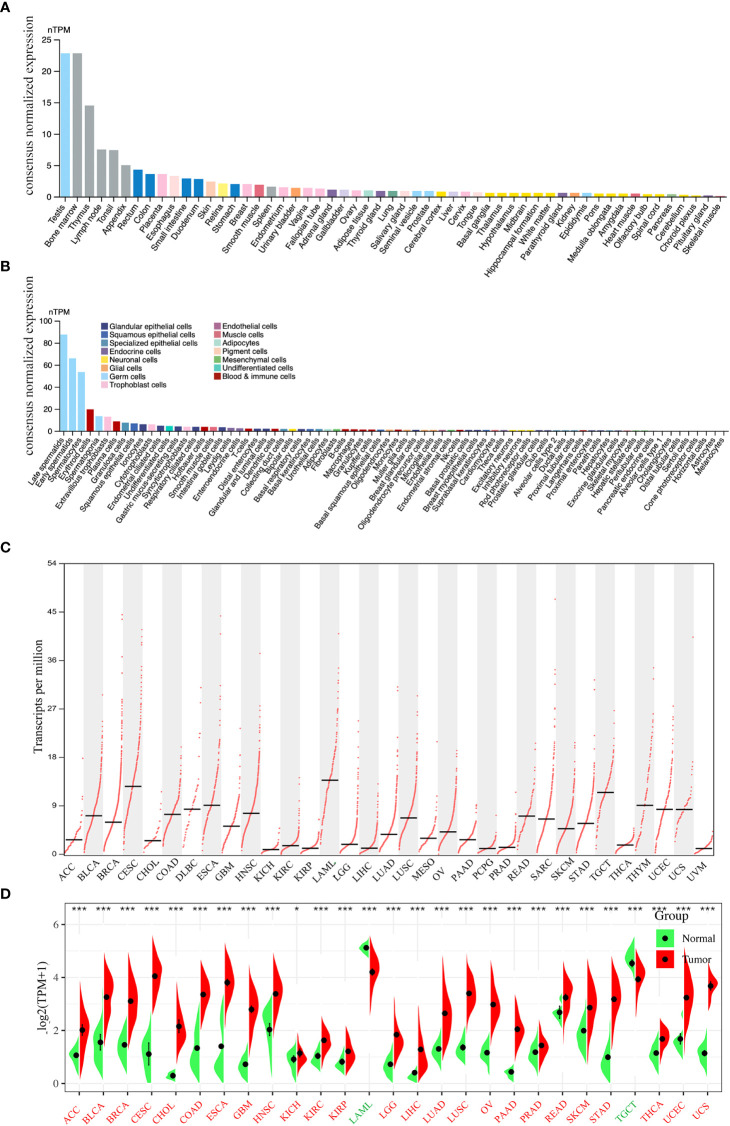
Expression levels of PLK4 mRNA in different tissues and cells. The expression status of the PLK4 mRNA in different normal tissues **(A)** and somatic cell types **(B)** were analyzed through Human Protein Atlas (HPA). **(C)** Based on the TCGA (The Cancer Genome Atlas) data, the expression levels of PLK4 mRNA in different cancer tissues were analyzed via GEPIA2. **(D)** The expression levels of PLK4 mRNA in tumors and normal tissues from TCGA and GTEx (The Genotype-Tissue Expression) databases were analyzed using SangerBox. *P < 0.05, ***P < 0.001.

The flowchart of this study is shown in **Figure 1**. We first analyzed PLK4 mRNA expression levels in somatic cells and normal tissues. As shown in **Figure 2A**, based on the data from HPA datasets, the expression level of PLK4 mRNA was the highest in testis, followed by the bone marrow and thymus. **Figure 2B** shows that among all cell types in the human body, PLK4 mRNA is expressed at the highest level in germ cells. PLK4 mRNA was detected in all somatic cells and normal tissues (all consensus normalized expression values >1). However, expression levels are low in most tissues and somatic cells, resulting in low tissue specificity.

Based on the data from various cancer types obtained from TCGA, we used the GEPIA2 program to analyze the differences in PLK4 mRNA levels. As shown in **Figure 2C**, TCGA database analyses of tumor tissues from 33 cancer types showed that PLK4 mRNA expression levels were different across tumor types and exhibit the highest level in acute myeloid leukemia (LAML). We then integrated data from normal tissues obtained from the GTEx database to further compare differences in PLK4 mRNA expression levels between normal tissues and tumor tissues. **Figure 2D** shows differences in the PLK4 mRNA expression levels between tumor and normal tissues. The expression levels of PLK4 mRNA in the tumor tissues of adrenocortical carcinoma (ACC), bladder urothelial carcinoma (BLCA), breast invasive carcinoma (BRCA), cervical squamous cell carcinoma and endocervical adenocarcinoma (CESC), cholangiocarcinoma (CHOL), colon adenocarcinoma (COAD), esophageal carcinoma (ESCA), glioblastoma multiforme (GBM), head and neck squamous cell carcinoma (HNSC), kidney chromophobe (KICH), kidney renal clear cell carcinoma (KIRC), kidney renal papillary cell carcinoma (KIRP), lower grade glioma (LGG), liver hepatocellular carcinoma (LIHC), lung adenocarcinoma (LUAD), lung squamous cell carcinoma (LUSC), ovarian serous cystadenocarcinoma (OV), pancreatic adenocarcinoma (PAAD), prostate adenocarcinoma (PRAD), rectum adenocarcinoma (READ), skin cutaneous melanoma (SKCM), stomach adenocarcinoma (STAD), thyroid carcinoma (THCA), uterine corpus endometrial carcinoma (UCEC), and uterine carcinosarcoma (UCS) are significantly higher than that in normal tissues (all *P* < 0.05).

Next, we analyzed PLK4 expression levels in different WHO grades, subtypes, and new types of gliomas. According to the World Health Organization (WHO) classification of Central Nervous System (CNS) tumors (WHO CNS5), gliomas can be divided into three types: oligodendroglioma with IDH mutation and 1p/19q deletion (mut+codel), astrocytoma with IDH mutation and 1p/19q non-codeletion (mut+non-codel), and glioblastoma with IDH-widetype. Patients with gliomas with IDH mutations and chromosome 1p/19q codeletions have a better survival outcome. Furthermore, promoter methylation status of the O6-methylguanine DNA methyltransferase (MGMT) is a prognostic indicator of the clinical response to treatment of glioblastoma patients with temozolomide (TMZ) (18). CGGA and TCGA database analyses showed that PLK4 expression levels were positively associated with glioma grades II, III, and IV (**Supplementary Figures 1A-C**). And ROC curves show the difference of PLK4 expression in LGG (WHO II and WHO III) and HGG (WHO IV) from TCGA, CGGA_693, and CGGA_325 databases (**Supplementary Figures 1D-F**). Moreover, the expression levels of PLK4 mRNA correlated with the WHO CNS5 types (**Supplementary Figure 1G-I**). The ROC curve showed that the expression level of PLK4 could distinguish between LGG (oligodendroglioma and astrocytoma) and HGG (gliomablastoma) in TCGA databases (**Supplementary Figure 1J-L**). We then analyzed the relationship between PLK4 expression, IDH gene mutations, 1p/19q codeletions, and methylation of MGMT promoters. According to the TCGA database and the CGGA_325, CGGA_693 datasets, patients with wild-type IDH, non-codeletion of chromosome 1p/19q, and unmethylated promoters of MGMT displayed significantly higher levels of PLK4 expression (**Supplementary Figure 1M-U**).

As a result of the TCGA’s findings, GBM can be divided into classical, mesenchymal, neural, and proneural subtypes based on gene expression data (19). GBM patients of classical or mesenchymal subtypes had worse prognosis. Based on the TCGA classification system, we annotated samples according to the four TCGA subtypes (**Supplementary Figures 1V-X**) and investigated whether PLK4 expression might be a marker of GBM subtypes. The expression of PLK4 was higher in classical or mesenchymal subtypes compared to neural or proneural subtypes in all three datasets. According to these results, PLK4 mRNA expression levels were higher in cancer tissues than in normal tissues. And in glioma, PLK4 mRNA were correlated with the grades and worse prognosis subtypes.

### PLK4 protein expression analysis data

**Figure 3:**
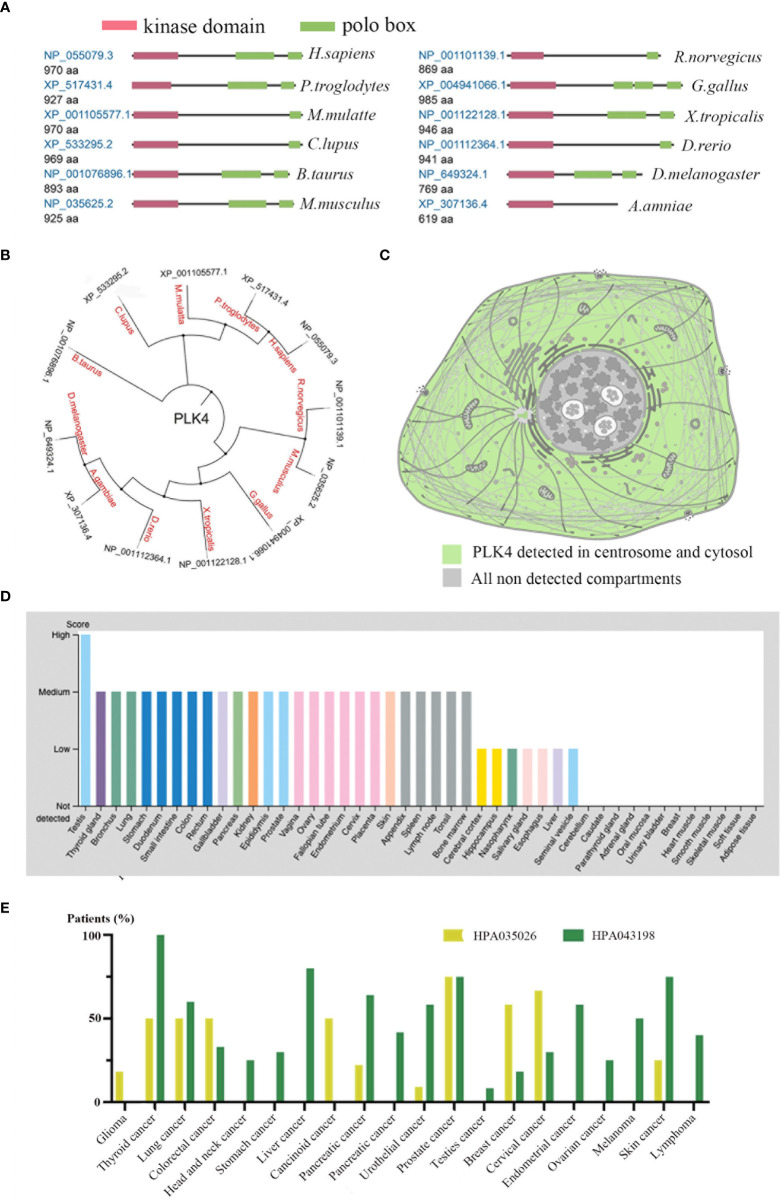
Expression levels of PLK4 protein in different normal tissues and tumors.**(A)** Conserved domains in the PLK4 protein structure among different species. **(B)** Phylogenetic tree for PLK4 protein across different species. **(C)** Subcellular localization of PLK4 protein in cells. **(D)** PLK4 protein expression levels across normal tissues were analyzed in HPA. **(E)** PLK4 protein expression levels in various cancer types were analyzed by immunohistochemistry staining using two antibodies (HPA035026, HPA043198) in HPA.

We initially examined PLK4 mRNA expression levels as described above. PLK4 is a protein kinase that phosphorylates various substrates at the protein level (20, 21). Therefore, we analyzed the protein expression levels of PLK4. As shown in **Figure 3A**, the protein structures of PLK4 appear to have been relatively conserved across different species during the evolutionary process (e.g., Homo sapiens, Canis lupus, Bos taurus). **Figure 3B** displays the phylogenetic tree obtained for the PLK4 protein among 12 species based on data obtained from the NCBI database. As shown in **Figure 3C**, PLK4 protein was localized to the cytoplasm and aggregated on centrosomes.

Next, we applied the HPA approach to analyze PLK4 protein levels in normal and tumor tissues separately. **Figure 3D** shows a comparison of PLK4 protein expression levels in different normal tissues, showing that PLK4 protein is expressed at the highest level in the testis. **Figure 3E** shows the expression levels of PLK4 protein in different cancers. Among the 20 cancer types examined in this study, more than half of patients with thyroid cancer, lung cancer, and prostate cancer showed medium to high levels of PLK4 protein expression in IHC staining results using two antibodies (HPA035026, HPA043198).

Consistent with the trend observed for PLK4 mRNA expression levels, the expression levels of PLK4 protein were also higher in proliferating tissues and cancer tissues.

### Survival analysis data

To better understand the impacts of PLK4 expression in various cancers, we analyzed the correlation between PLK4 expression levels and the prognosis of patients with different tumors. We ranked the patients’ PLK4 mRNA levels from high to low and divided them into two groups: the PLK4 high expression group and the PLK4 low expression group.

**Figure 4:**
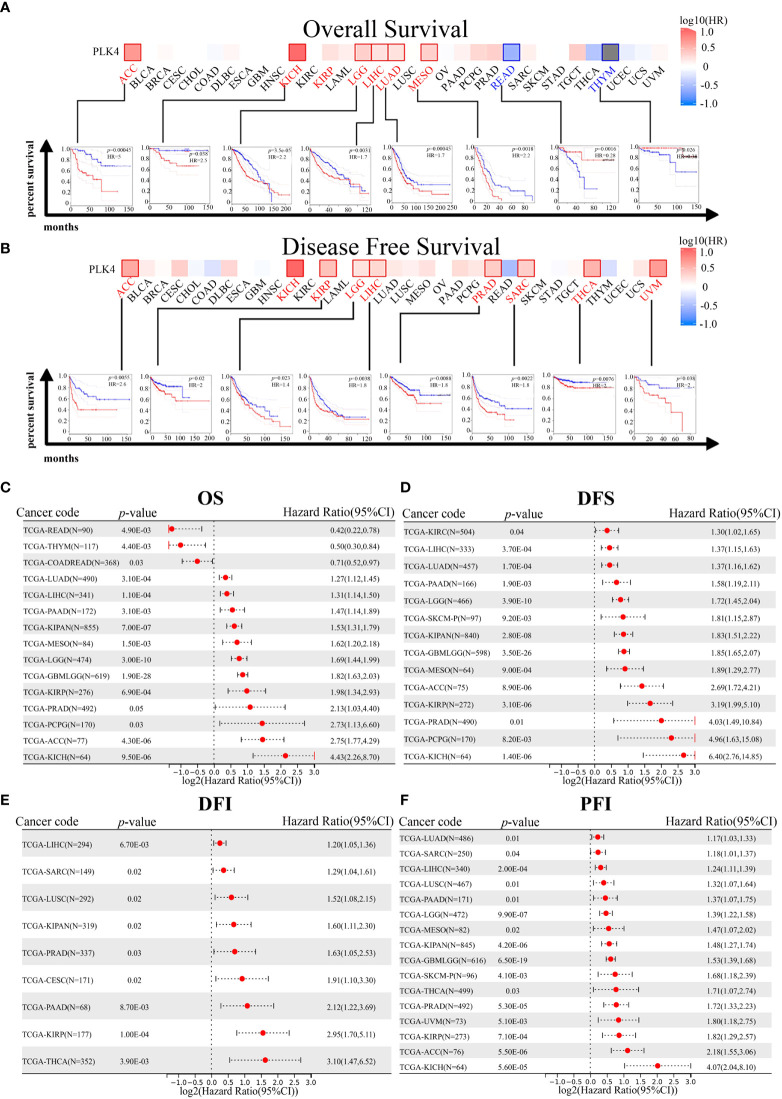
Correlation between PLK4 expression and survival prognosis for various cancers in TCGA. We analyzed overall survival (OS) **(A)** and disease-free survival (DFS) **(B)** using the log-rank test in different tumor types from TCGA. A survival map with positive results is shown. The correlation between *PLK4* expression and overall survival (OS) **(C)**, disease free survival (DFS) **(D)**, disease free interval (DFI) **(E)**, and progression free interval (PFI) **(F)** in different cancer types using Cox regression survival analysis for TCGA datasets.

Prognostic analysis using the GEPIA database also indicated that high PLK4 levels in a variety of tumors, including ACC, KICH, LGG, and LIHC, were associated with poor OS and recurrence-free survival (RFS) (**Figures 4A, B**). Consistently, prognostic cox regression analysis using the TCGA database also indicated that high PLK4 levels in a variety of tumors, including LUAD, LIHC, pan-kidney cohort (KIPAN), mesothelioma (MESO), LGG, glioma (GBMLGG), KIRP, PRAD, pheochromocytoma and paraganglioma (PCPG), ACC, and KICH, were associated with OS, DFS, disease free interval (DFI) and progression free interval (PFI) (**Figures 4C–F**). These findings suggest that PLK4 is an oncogene associated with poor prognosis in multiple tumors, including glioma.

TGGA and CGGA datasets for patients with gliomas indicated a correlation between PLK4 expression and prognosis. As shown in the TCGA, CGGA_693, and CGGA_325 datasets, patients with pan-glioma and LGG had shorter overall survival when PLK4 expression levels were high (**Supplementary Figures 2A–I**). For the purpose of calculating AUC, R package pROC (version 1.17.0.1) was used. Furthermore, the ROC curve demonstrated that PLK4 could be useful in predicting the prognosis of pan-glioma based on the CGGA_325, CGGA_693, and TCGA datasets (**Supplementary Figures 2J–L**). A high level of PLK4 expression in the TCGA dataset was associated with shorter disease-specific survival (DSS) among pan-glioma and LGG patients, as well as shorter progression-free survival (PFS) among these patients (**Supplementary Figures 2M–R**). In Cox regression analyses of the TCGA, CGGA_325, and CGGA_693 databases, tumor grade, chemotherapy, IDH mutation status, and 1p/19q codeletion were found to represent independent prognostic factors (**Supplementary Figures 3A, C, E**). Several independent prognostic factors were incorporated into the nomograms, namely tumor grade, expression levels of PLK4, IDH mutation status, 1p/19q codeletion status, and the MGMT promoter status (**Supplementary Figures 3B, D, F**).

Based on TGGA and CGGA datasets, we then examined the relationship between PLK4 expression levels and prognosis of patients with oligodendrogliomas, astrocytomas, and glioblastomas. The TCGA and CGGA_693 datasets showed that the prognosis of patients with oligodendroglioma with PLK4-high levels was worse than that of patients with PLK4-low levels (**Supplementary Figures 4A-C**). According to the CGGA_693 and CGGA_325 datasets, the prognosis of patients with astrocytomas in the PLK4-high group was worse than that of patients in the PLK4-low group (**Supplementary Figures 4D–F**). The survival rate of glioblastoma patients in PLK4-high groups was worse than that of patients in PLK4-low groups in the CGGA_693 and CGGA_325 datasets (**Supplementary Figure 4G–I**). The prognosis of patients in the PLK4-high group was worse than that of patients in the PLK4-low group, regardless of the IDH status, in the CGGA_325 and CGGA_693 datasets (**Supplementary Figures 5A-C**). Only in 1p19q non-codeletion glioma patients in the CGGA_325, CGGA_693, and TCGA datasets in the PLK4-high group have a worse prognosis than those in the PLK4-low group (**Supplementary Figures 5D-F**). Both in the CGGA_325, CGGA_693, and TCGA datasets, patients with PLK4-high glioma had worse prognoses than those with PLK4-low glioma (**Supplementary Figures 5G–I**). A poor prognosis was observed in PLK4-high glioma patients compared to those in PLK4-low glioma patients in CGGA_325 and CGGA_693 datasets, both of which were treated with chemoradiotherapy and non-chemoradiotherapy (**Supplementary Figures 5J-M**). There was an overall association between PLK4 mRNA expression levels and prognosis for multiple cancers according to the results of the study. Additionally, patients with glioma who expressed high levels of PLK4 had a poorer prognosis.

### Enrichment analysis of PLK4-related partners

**Figure 5:**
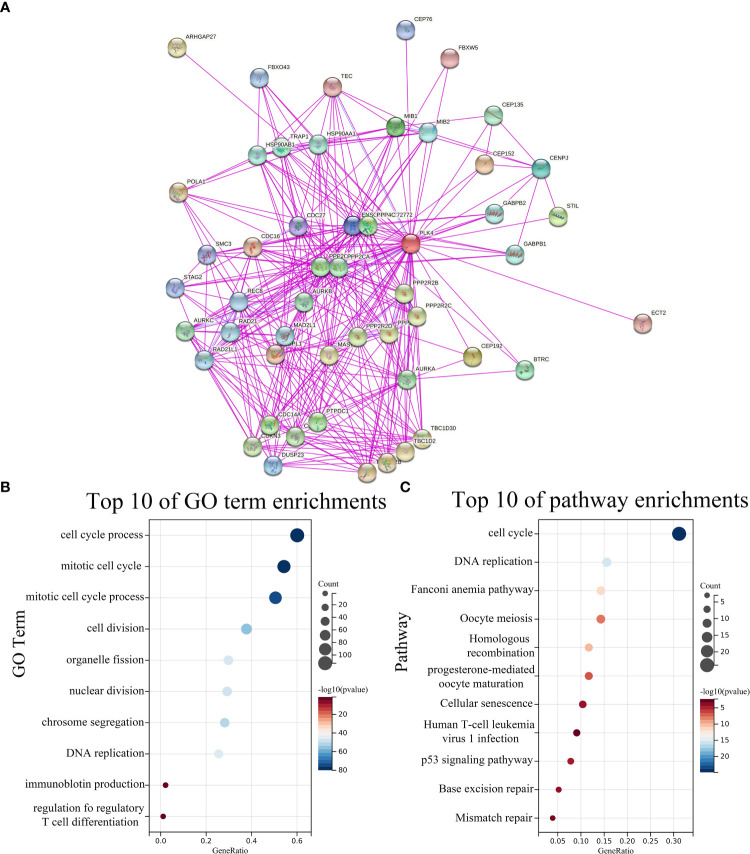
PLK4-related genes enrichment analysis. **(A)** PLK4-binding proteins obtained using the STRING tool. Significantly enriched GO annotations **(B)** and KEGG pathways **(C)** of 100 genes positively associated with PLK4 expression in all TCGA tumors.

To further investigate the underlying molecular mechanisms through which PLK4 drives tumorigenesis, we explored PLK4-related genes and performed a series of GO and KEGG enrichment analyses on these genes. We identified 50 PLK4-binding proteins supported by experimental evidence using the STRING tool. The protein–protein interaction network for these proteins was shown in **Figure 5A**. We then obtained the top 100 genes positively associated with PLK4 expression using the GEPIA2 tool combined with all TCGA tumor expression data. Then we performed GO and KEGG enrichment analysis using Sangerbox enrichment analysis on above 100 genes. GO analysis showed that these genes are significantly enriched in “cell cycle”, “T cell differentiation” and “chromosome segregation” et al. functions (**Figure 5B**). And KEGG pathway analysis showed enrichment in the “cell cycle”, “P53 signaling pathway” et al. pathways (**Figure 5C**). The GO and KEGG enrichment analysis indicate that PLK4 were closely involved in tumor immune microenvironment, genome instability, and cell cycle progression in pan-cancer.

To further validate our findings in gliomas, we used glioma data from TCGA, CGGA_693 and CGGA_325 databases for GO and KEGG enrichment analyses (**Supplementary Figure 6**). The heat maps of PLK4-related genes were shown in **Supplementary Figures 6A, D, G**. In TCGA, CGGA_693 and CGGA_325 databases, we selected genes whose absolute value of correlation with PLK4 was greater than 0.35, and then analyzed these genes by Sangerbox enrichment analysis tool for GO and KEGG enrichment. GO analysis shows that these PLK4-related genes are mainly enriched in “cell cycle”, “T cell mediated immune response to tumor cell”, “cell division” and “T cell receptor signaling pathway” (**Supplementary Figures 6B, E, H**). KEGG analysis of glioma database showed that the PLK4-related gene is mainly enriched in “P53 signaling pathway”, “T cell and B cell receptor signaling pathways”, “cell cycle”, “mismatch repair” and “PD-L1 expression and PD-1 checkpoint pathway in cancer” (**Supplementary Figures 6C, F, I**).

Additionally, we downloaded the GSE180958 database uploaded by Yang et al. who successfully established PLK4 knockdown cell lines of bladder cancer cell, followed by RNA-seq analysis. We considered a 1.5-fold change of expression and *P* < 0.05 as the standard from RNA-seq data of 5637 to select 1245 differentially expressed genes (DEGs). And the volcano plot of PLK4 related genes was shown in **Supplementary Figure 6J**. Functional profiling analysis suggested that the affected genes were mainly enriched in biological processes including B cell proliferation, T cell activation, inflammatory response functions and IL-17 signaling pathway (**Supplementary Figures 6K, L**).

Therefore, PLK4 potentially regulates tumor immune microenvironment, cell cycle progression, and genome instability in multiple cancers, especially glioma.

### PLK4 regulates tumor infiltration of immune cells, and tumor immune microenvironment in gliomas and other cancer types

**Figure 6:**
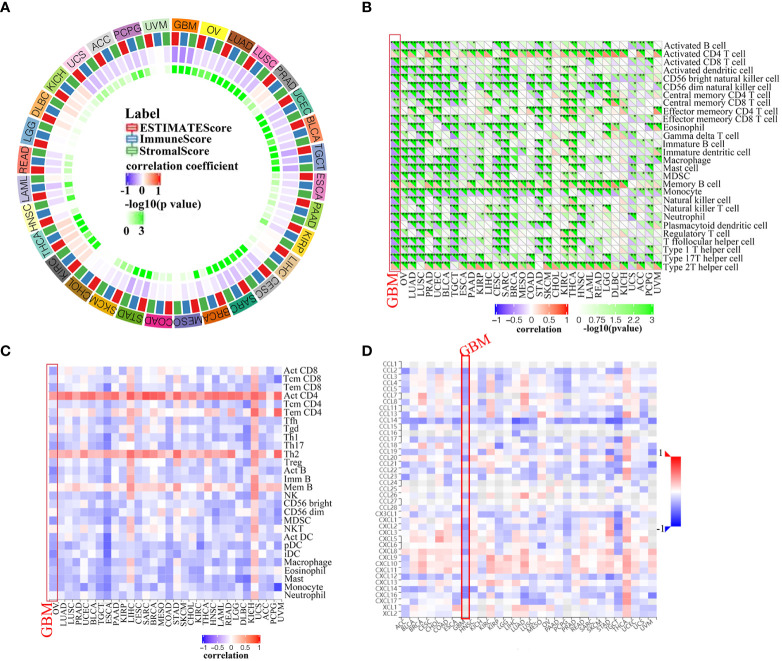
Correlation analysis between PLK4 expression and immune infiltration levels. **(A)** Analyzing the correlation between *PLK4* expression and ESTIMATE scores in various tumors was performed by Sangerbox. **(B)** According to the Sangerbox website, *PLK4* expression correlates with immune cell infiltration in various types of tumors. *P<0.05, **P<0.01, ***P<0.001, **(C)** An analysis of the relationship between *PLK4* expression levels in tumor tissues and abundance of tumor-infiltrating lymphocytes (TILs) was performed using the online tool TISIDB. **(D)** Analysis of the relationship between *PLK4* expression levels in tumor tissues and chemokines was performed using the online tool TISIDB.

The tumor immune microenvironment (TIME) is comprised of tumor infiltrating immune cells (TIICs), which are involved in tumor biogenesis and metastasis. Furthermore, the number of tumor-infiltrating lymphocytes (TILs) has been implicated as a critical determinant of sentinel lymph node status and survival (22). Therefore, PLK4 expression levels and TIIC composition were investigated in various tumors based on the enrichment analysis above. Firstly, we determined whether PLK4 expression in pan-cancers was correlated with the ESTIMATE scores (ESTIMATE, immune, and stromal scores). Immune scores reflect the percentage of immune cells infiltrated by the tumor; stromal scores reflect the percentage of stromal cells; estimates scores reflect the status of the tumor’s immune microenvironment and the purity of the tumor. ESTIMATE, immune, and stromal scores correlate negatively with PLK4 expression in most TCGA cancers (**Figure 6A**). As a result of high PLK4 expression, immune and stromal cells were less likely to infiltrate into several tumors, resulting in a higher tumor purity. ESTIMATE score, Immune score, and Stromal score are negatively correlated with PLK4 mRNA expression level in GBM (**Supplementary Figure 7**). Based on the Sangerbox (**Figure 6B**) and TISIDB (**Figure 6C**) databases, we examined the relationship between PLK4 expression and TIIC levels in various tumor types. Multiple immune cell types were observed to be negatively correlated with PLK4 expression in various cancer types, such as GBM, lung adenocarcinoma (LUAD), prostate adenocarcinoma (PRAD), skin cutaneous melanoma (SKCM), etc. TCGA, CGGA_693, and CGGA_325 datasets showed negative correlations between PLK4 mRNA levels and multiple TIICs/TILs (**Supplementary Figure 8**). A significant proportion of GBM patients are sensitive to immunotherapy as a result of PLK4 expression, which modulates immune cell recruitment. Then, we found that PLK4 was negatively correlated with chemokines, indicating the potential mechanism of reduced immune cell recruitment (**Figure 6D**).

We then further investigated the effects of PLK4 expression on the inflammatory status of GBM by screening seven metagenes, namely, *HCK, IgG, Interferon, LCK, MHC-I, MHC-II*, and *STAT1 (* 23, 24). Our results showed lower enrichment scores for multiple metagenes such as *LCK*, *MHC-II* and *STAT1* in GBM patients with higher PLK4 expression levels from the TCGA, CGGA_693, and CGGA_325 datasets (**Supplementary Figure 9**). This suggested that a correlation was observed between inflammatory signatures and PLK4 in GBM.

Totally, despite the fact that the expression levels of PLK4 in gliomas and other cancer types varied, we found that they modulated infiltration of immune cells, and the tumor immune microenvironment in general.

### Single-cell sequencing investigated the correlation between PLK4 and cell clusters

To further reveal the correlation between PLK4 and immune infiltration at the level of individual cells, the scRNA-seq analysis was performed for exploring the PLK4 expression level in different cell types among TME in gliomas. Firstly, the R package “Seurat” was employed to process the single-cell data expression matrix, by which we annotated seven gliomagenesis-associated cell clusters, including myeloid cells, neoplastic cells, oligodendrocyte precursor cells, oligodendrocytes, astrocytes, neurons, and vascular cells. (**Supplementary Figure 10A**). Then, we evaluated the PLK4 expression in all clusters and found that PLK4 was mainly expressed in myeloid cells and neoplastic cells (**Supplementary Figure 10B**). So, we further annotated the myeloid cells into M1 macrophages and M2 macrophages (**Supplementary Figure 10C**). We then performed pseudotime trajectory analyses using the “Monocle” R package on neoplastic cells and macrophages. There are four main branches and three branch points in neoplastic cells, and there are seven different states in the cells. The main and branch points of the M1 macrophages were identified, and 13 states of the cells were identified. Moreover, PLK4 expression was significantly increased in state six, state three, and state five of neoplastic cells, whereas PLK4 expression was slightly elevated in states ten and three of M1 macrophages. Overall, PLK4 was mainly expressed in myeloid cells and neoplastic cells and exhibited a relatively stable expression in nearly all developmental stages (**Supplementary Figures 10D, E**).

### The PLK4 gene is altered in several types of cancer, including gliomas, and is associated with the progression of those tumors

**Figure 7:**
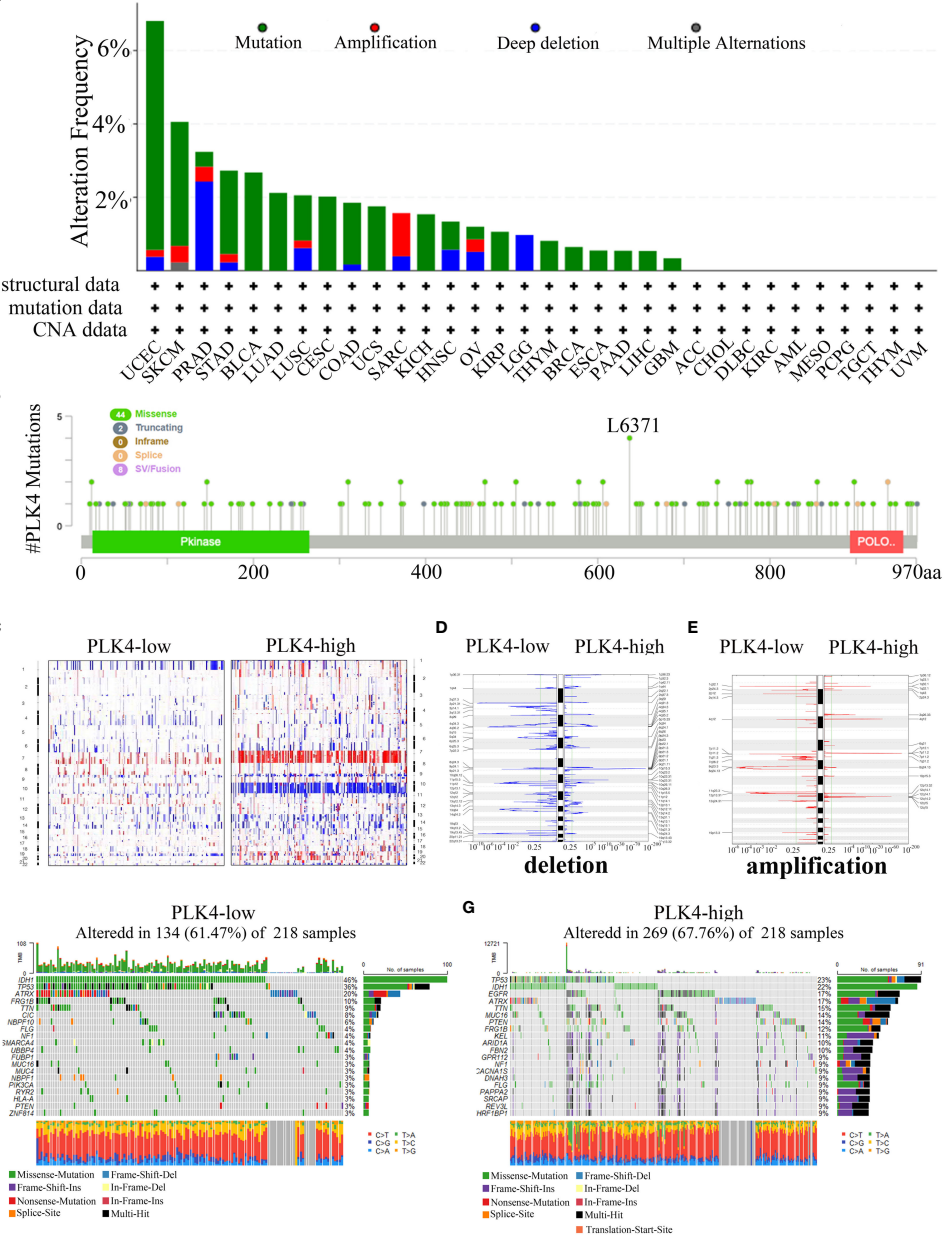
Analyzing complex cancer genomics and clinical profiles in relation to PLK4 expression and distinguishing certain genomic profiles associated with the expression of PLK4. **(A)** Mutation rate of *PLK4* gene in various tumors. **(B)** By using the cBioPortal tool, the mutation sites of *PLK4* have been identified in multiple tumor types. **(C)** CNA profiles regarding high and low levels of expression of *PLK4*. **(D, E)** An analysis of the frequency of amplifications and deletions in gliomas with high and low expression levels of the *PLK4* gene (Blue, deletion; red, amplification). **(F, G)** A comparative analysis of glioma somatic mutations (25% *PLK4* low and 25% PLK4 high).

Oncogenes and tumor suppressor genes are affected by genetic changes resulting in mutations, deletions, or amplifications, which can lead to tumor progression (25). Then, we used the cBioPortal portal to analyze TCGA cancer datasets for different types of alterations in the PLK4 gene. PLK4 gene mutation(>6%) are most commonly observed in the UCEC (**Figure 7A**). The PLK4 gene is most commonly mutated in cancers through missense mutations, according to the cBioPortal database analysis (**Figure 7B**).

Our gene enrichment analysis indicates that PLK4 plays a key role in genomic instability. The TCGA glioma dataset was then analyzed to determine whether PLK4 expression is associated with somatic mutations and copy number variations (CNVs). As shown in **Figure 7C**, the CNV profiles of the PLK4^low^ (n = 166) and PLK4^high^ (n = 166) samples were compared. A number of deletions and amplification peaks were observed in the PLK4^low^ samples in the 2q37.3 chromosomal loci and 19p13.43 chromosomal loci, respectively. There were deletions observed in the 9p21.1 chromosome as well as amplifications in the 7p11.2, 1q32.1, and 12q14.1 chromosomes of the PLK4^high^ samples (**Figures 7D, E**). According to PLK4^low^ (n = 166), the IDH1 gene had 46% mutations, TP53 36% mutations, and ATRX 20% mutations, whereas PLK4^high^ (n = 166) had 23% such mutations, IDH1 22% mutations, and EGFR 17% mutations. (**Figures 7F, G**).

Overall, these results showed the gene mutations, amplifications, and deletions of PLK4 were widespread in multiple tumors. It was found that missense mutations were the most common genetic alteration in various cancers. The expression levels of PLK4 in glioma tissues revealed distinct somatic mutations and copy number variations. Based on these findings, it is suggested that mutations in the PLK4 gene may be involved in the induction of genomic instability and the progression and growth of various tumors, particularly gliomas.

### PLK4 is highly expressed in glioma, and is closely related to cell cycle, cell proliferation

After completing the enrichment analysis results presented in **Figure 5** and **Supplementary Figure 6**, we carried out an *in vitro* and *in vivo* validation test. We collected protein and RNA samples from multiple glioma cell lines (U87, LN229, U251MG, A172, and T98) and human astrocyte (HA), and examined the mRNA and protein levels of PLK4 (**Supplementary Figures 11A, B**). Compared with HA cells, the glioma cell lines had significantly higher mRNA and protein levels of PLK4; among the glioma lines, U87 and LN229 had the highest expression levels of PLK4 mRNA and protein. Previous studies of our group found that PLK4 RNA expression in HGG patients was higher than that in LGG patients (5). To determine whether PLK4 was involved in glioma progression, we decreased PLK4 expression in U87 and LN229 cells by transfecting the cells with two siRNAs (si-PLK4#1 and si-PLK4#2) (**Supplementary Figure 11C**). Propidium iodide staining was used to detect the effect of PLK4 knockdown on the DNA content of glioma cells by flow cytometry. PLK4 knockdown increased >4N (polyploid) DNA content in U87 (**Supplementary Figures 11D, E**) and LN229 (**Supplementary Figures 11F, G**) cell lines. These increases indicated that PLK4 knockdown resulted in defective mitosis and significantly impaired the cell cycle. In addition, decreased PLK4 expression caused significant reduction in cell viability. Cell proliferation was also retarded by decreased PLK4 expression, as shown by the results of EdU and CCK8 assays in U87 (**Supplementary Figures 11H, J**) and LN229 (**Supplementary Figures 11I, K**) cell lines.

Together, these results proved that PLK4 was highly expressed in glioma. Moreover, consistent with findings from the GO and KEGG analyses, PLK4 knockdown significantly impeded the cell cycle and slowed cell proliferation. Thus, PLK4 promotes the malignant phenotype of glioma by regulating cell cycle and cell proliferation, providing a new idea for us to explore novel therapeutic targets for glioma.

### PLK4 promotes immunosuppressive tumor microenvironment by impairing M1-macrophage infiltration

**Figure 8:**
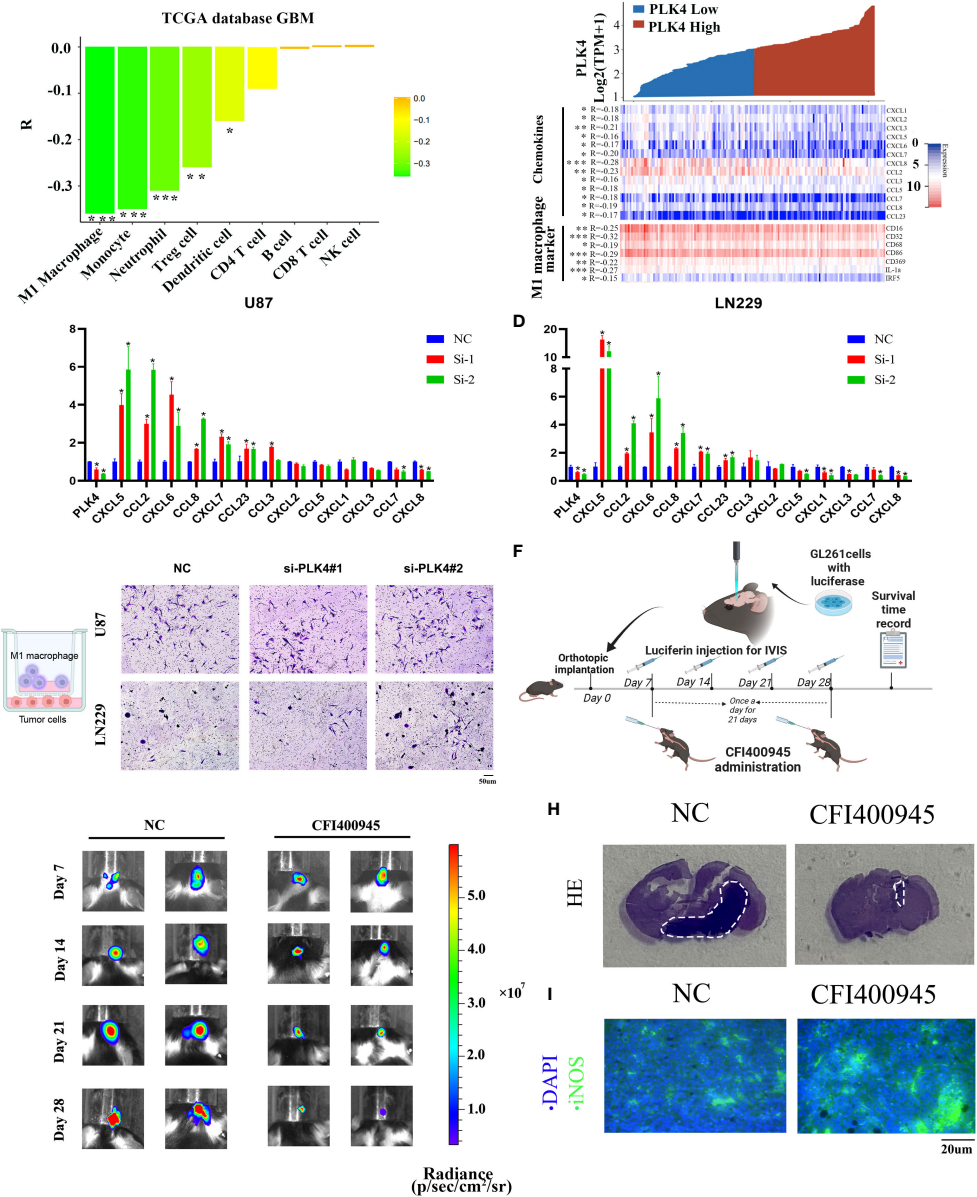
PLK4 is correlated with M1 macrophage infiltration in glioma. **(A)** Analysis of immune cell infiltration and *PLK4* expression in TCGA-GBM data sets by quanTIseq algorithm. *P<0.05, **P < 0.01, ***P < 0.001. **(B)** The relationship between PLK4 mRNA expression and M1 macrophage-related chemokines/markers in GBM. *P<0.05, **P < 0.01, ***P < 0.001 **(C, D)** The expression levels of several M1 macrophage-related chemokines after knockdown of *PLK4* in U87 and LN229 cells. * *P *< 0.05. **(E)** Infiltration of M1 macrophages in NC (left), si-PLK4#1 (middle), si-PLK4#2 (right) of U87 and LN229 cells. **(F)** Schematic illustration of the experimental design for orthotopic model construction. **(G)** Imagining of GBM tumor-bearing mice and corresponding quantification of ROI signal of the tumor site at 7, 14, 21 and 28 days. **(H)** HE staining showed decreased tumor volume in mice with CFI400945 administration. **(I)** Immunofluorescence staining showed M1 macrophages infiltration of tumor region in mice.

To further explore the impact of PLK4 on immune cells infiltration, we evaluated the classical proportions of 9 types of infiltrating immune cells (M1 macrophages, B cells, Treg cells, CD8 T cells, CD4 T cells, NK cells, monocytes, dendritic cells, neutrophils) using the quanTIseq algorithm by Spearman’s rank correlation test in TCGA-GBM datasets. According to the results of quanTIseq algorithm, PLK4 exhibited markedly negative correlation with M1 macrophage infiltration (**Figure 8A**). In order to further confirm this conclusion, we drew a heatmap of the expression of M1 macrophage-related chemokines and markers in the PLK4 high and low expression groups. Similarly, the expression levels of M1 macrophage-related chemokines and markers were relatively low in the PLK4 high expression group (**Figure 8B**). Additionally, the above chemokines and maker genes were screened and validated *via* RT-qPCR. RT-qPCR results show that CXCL5, CCL2, CXCL6, CCL8, CXCL7, CCL23 are upregulated after PLK4 knockdown in both LN229 and U87 cells (**Figures 8C, D**). Consistently, silencing of PLK4 in U87 cells significantly increased the infiltration of M1 macrophages (**Figure 8E**). Then, the orthotopic model was established to verify the immune role of PLK4 (**Figure 8F**). Living imaging and HE staining results showed that the tumor growth of CFI400945-treated mice was significantly inhibited (**Figures 8G, H** and **Supplementary Figure 12**). The results of immunofluorescence staining showed that the tumor tissue of CFI400945-treated mice exhibit increased M1 macrophages infiltration (**Figure 8I**). As mentioned above, PLK4 may contribute to the immunosuppressive microenvironment by inhibiting the expression of chemokines and thereby impairing the recruitment of M1 macrophages.

## Discussion

The PLKs comprise a protein family that has been identified as critical for spindle formation (26), centrosome duplication (27), and mitotic entry (28). PLK4 is the most structurally differentiated member of the PLK family, and its C-terminal noncatalytic region contains only one polo-box (PB) domain. PLK4 localizes to the centrosome and allows mitosis to proceed correctly *via* tight control of centriole duplication (24). In this study, evolutionary analysis of the PLK4 protein across multiple species (**Figures 3A, B**) showed that the PLK4 protein structure is highly conserved across different species, indicating potentially similar physiological mechanisms for PLK4 activity across species. Previous studies have identified associations between PLK4 and various diseases, especially cancers. Whether PLK4 plays an important role in the occurrence and development of various tumors *via* underlying molecular mechanisms is worth exploring. We found few publications investigating the role of PLK4 from a pan-cancer perspective. Using TCGA, CGGA, and GEO databases, we comprehensively investigated the functional role of PLK4 in multiple tumors, especially gliomas.

A comprehensive analysis of patient data from various databases was conducted in this study in order to investigate the role of PLK4 in multiple cancers. Multiple types of cancer, including gliomas, showed increased levels of PLK4 mRNA and protein (**Figure 2**). There is a correlation between PLK4 expression in gliomas and tumor grades, subtypes, and new WHO types of gliomas (IDH mutation +1p19q codel, IDH mutation +1p19q non-codel, and IDH wild type) (**Supplementary Figures 1A–L**). PLK4 expression was associated with tumor prognosis according to the pan-cancer analysis (**Figure 4**). High PLK4 expression was associated with clinical characteristic such as IDH wildtype status, 1p/19q non-codeleted status, and demethylation of MGMT, all of which were correlated with poor prognosis in patients with glioma (**Supplementary Figures 1M-U**). The results of the Cox regression analysis indicated that PLK4 could serve as an independent prognostic indicator in patients with gliomas (**Supplementary Figure 3**). The expression of PLK4 in neuroblastoma (NB) is associated with a poor prognosis and has been reported in both primary and metastatic forms, suggesting that PLK4 may accelerate tumorigenesis in NB (29). The expression of PLK4 in breast cancer tissues has also been found to be overexpressed (30), which was associated with poor prognosis and aggressiveness (31, 32). Overall, our analysis showed that PLK4 was a prognostic biomarker in pan-cancers and accounted for unfavorable survival outcomes in gliomas.

Secondly, in order to reveal the oncogenic mechanisms of PLK4 in pan-cancer, the top 100 PLK4 positively correlated genes in pan-cancer were collected for enrichment analysis (**Figure 5**). Similar to our findings in pan-cancer, PLK4-related genes showed enrichment in pathways related to immune, genetic, and cell cycle functions in glioma (**Supplementary Figure 6**). Consequently, we were primarily interested in the molecular mechanisms by which PLK4 regulates immune responses, genetic changes, and cell cycle regulation.

Tumor immunosurveillance and therapeutic response are both regulated by TIICs in the tumor microenvironment (TME) (33). PLK4 expression contributes to the activation of metabolic and immune signaling pathways in various tumor types according to GO and KEGG pathway enrichment analyses (**Figure 5** and **Supplementary Figure 6**). To determine whether there is a relationship between the expression levels of PLK4 and the tumor immune microenvironment (TIM), we explored the relationship further. A research study conducted by ESTIMATE demonstrated a correlation between high PLK4 expression and immune cell and stromal cell infiltration across a wide range of cancers (**Figure 6A**). Based on these results, PLK4 seems to inhibit the infiltration of immune cells into TME, allowing tumor cells to evade the immune system. GBM patients’ responses to immunotherapy may be affected by PLK4 expression, which was negatively correlated with immune cell infiltration (**Figure 6** and **Supplementary Figure 8**).

Somatic mutations might potentiate the transformation of normal cells to tumor cells. Cancer cells with somatic mutations are immune evading and do not respond well to treatment (34). PLK4 gene alterations across several cancer types were primarily comprised of amplifications, mutations, and deep deletions (**Figure 7A**). The results of this study showed that gliomas with low expression of PLK4 had a higher number of IDH1 mutations (46%) as compared to those with high expression of PLK4 (22%) (**Figures 7F, G**). According to previous reports, LGG patients had a higher survival rate than those with HGG, and IDH mutations were more common in LGG patients. Patients with low PLK4 expression gliomas also had a higher TP53 mutation frequency (36% vs. 23%) than patients with high PLK4 expression gliomas (**Figures 7F, G**). The TP53 gene is well known as a tumor suppressor that inhibits the development of GBM. Therefore, PLK4 may serve as a valuable prognostic biomarker in the treatment of glioblastoma and other types of cancer.

According to the results of qRT-PCR, PLK4 exhibited a high expression level in glioma tissues and cell lines (**Supplementary Figures 11A, B**). The results of flow cytometry, CCK8 and EdU assays showed that PLK4 can accelerate cell cycle and promote cell proliferation in glioma cell lines (**Supplementary Figures 11D-K**). Immune infiltration analysis showed that PLK4 was inversely correlated with infiltration of M1 macrophages (**Figures 8A, B**). Consistently, we found that PLK4 might impair the infiltration of M1 macrophage by blocking the expression of M1-related chemokines, including CXCL5, CCL2, CXCL6, CCL8, CXCL7, CCL23 (**Figures 8C, D**). Transwell experiments demonstrated that infiltration of M1 macrophages was increased after knockdown of PLK4 (**Figure 8E**). Then orthotopic model was established for further verification of the carcinogenic function of PLK4 (**Figure 8F**). The PLK4 inhibitor CFI400945 was administered to mice, followed imaging and HE staining showed that CFI400945 can significantly inhibit tumor progression (**Figures 8G, H**). Immunofluorescence staining confirmed the suppressive role of PLK4 to M1 macrophages infiltration (**Figure 8I**). Therefore, PLK4 might serve as a promising biomarker in predicting immunotherapeutic responses, sparking new hope for patients suffering glioma.

Accordingly, our results indicate that PLK4 is aberrantly expressed in a wide range of cancers, and influences cancer patients’ prognoses. A wide variety of cancer types were found to harbor mutations, duplications, and amplifications of the PLK4 gene. The overexpressed PLK4 prompts cell cycle and cell proliferation. Besides, a significant correlation has been found between PLK4 expression and the infiltration of immune cells and the response to immunotherapy in multiple cancers. *In vitro* and *in vivo* experiments confirmed that PLK4 functioned as an oncogene and was associated with M1 macrophage infiltrations in gliomas. Totally, our study suggested that PLK4 initiates crosstalk between cell cycle, cell proliferation and M1 macrophage infiltration, contributing to malignant progression in gliomas. Meanwhile, PLK4 might serve as a vulnerability that can be targeted to overcome gliomas.

## Data availability statement

The original contributions presented in the study are included in the article/**Supplementary Materials**. Further inquiries can be directed to the corresponding authors.

## Ethics statement

The animal study was reviewed and approved by Animal Ethical and Welfare Committee of Tianjin Medical University.

## Author contributions

LH and YL conceived the article. XZ, ZL and CW drafted the manuscript and revised it before submission. CW, LL and JZ collected the references. ZL, XZ and SL performed the experiments. LL and HL contributed reagents/materials/analysis tools. All authors contributed to the article and approved the submitted version.

## Supplementary material

Supplementary Figure 1 PLK4 expression signature in CGGA and TCGA databases. The expression level **(A-C)** of PLK4 in WHO II, WHO III, and WHO IV glioma and ROC curve **(D-F)** analysis via TCGA, CGGA_325, and CGGA_693 databases. The expression level **(G-I)** of PLK4 in oligodendroglioma, astrocytoma, and glioblastoma and ROC curve **(J-L)** analysis via TCGA, CGGA-325, and CGGA-693 databases. The expression level of PLK4 in different IDH status **(M-O)**, 1p/19q status **(P-R)** and MGMT promotor status **(S-U)** via TCGA, CGGA_325, and CGGA_693 databases. The expression level **(V-X)** of PLK4 in glioma subtypes via TCGA, CGGA-325, and CGGA-693 databases. **P*<0.05, ***P*<0.01, ****P*<0.001, *****P*<0.0001.

Supplementary Figure 2 Relationship between expression of PLK4 and the survival of patients with glioma in CGGA and TCGA databases. The relationships between the expression of PLK4 and prognosis in pan-glioma **(A-C)**, lower grade glioma (LGG) **(D-F)** and glioblastoma (GBM) **(G-I)** by TCGA, CGGA_325, and CGGA_693 databases. Receiver operating characteristic (ROC) curve to assess sensitivity and specificity of PLK4 expression as a prognostic biomarker for pan-glioma **(J-L)**. The relationship between PLK4 expression and DSS of pan-glioma, LGG, and GBM in TCGA databases **(M-O)**. The relationship between PLK4 expression and PFI of pan-glioma, LGG, and GBM in TCGA databases **(P-R)**. **P*<0.05, ****P*<0.001.

Supplementary Figure 3 An analysis of PLK4 and clinical features of gliomas using univariate and multivariate Cox models. Multivariate Cox analyses of PLK4 and clinical features of glioma in TCGA **(A)**, CGGA_693 **(C)**, and CGGA_325 **(E)** databases. The nomogram model of PLK4 and clinical features in TCGA **(B)**, CGGA_693 **(D)**, and CGGA_325 **(F)** databases. **P*<0.05, ***P*<0.01, ****P*<0.001, *****P*<0.0001.

Supplementary Figure 4 Relationship between expression of PLK4 and the survival of patients with glioma in CGGA and TCGA databases. The relationships between the expression of PLK4 and prognosis in oligodendroglioma **(A-C)**, astrocytoma **(D-F)** and glioblastoma **(G-I)** by TCGA, CGGA_693, and CGGA_325 databases.

Supplementary Figure 5 Prognosis analyses of glioma patients. Kaplan-Meier analyses of overall survival among four patient groups stratified by the PLK4 mRNA level and IDH status **(A-C)**, 1p/19q status **(D-F)**), MGMT promotor status **(G-I)**, radiotherapy **(J, K)**, and chemotherapy **(L, M)** in TCGA, CGGA_693 and CGGA_325. **P*<0.05, ***P*<0.01, ****P*<0.001, *****P*<0.0001.

Supplementary Figure 6 Functional enrichment analysis of PLK4-related genes in glioma database. The heat map **(A, D, G)** and enrichment analysis **(B, C, E, F, H, I)** of the genes correlated with PLK4 in glioma in TCGA, CGGA_693, and CGGA_325 datasets. The volcano figure **(J)** and enrichment analysis **(K, L)** of the genes correlated with PLK4 in bladder cancer in GSE180958 datasets. **P*<0.05, ***P*<0.01, ****P*<0.001.

Supplementary Figure 7 The expression of PLK4 mRNA in GBM and the ESTIMATE Score are correlated. The correlations between PLK4 mRNA expression level and ESTIMATE Score (*P*=2.4e-9, R=-0.46) **(A)**, Immune Score (*P*=1.1e-9, R=-0.47) **(B)** and Stromal Score (*P*=1.6e-7, R=-0.41) (**C**) of GBM in TCGA dataset.

Supplementary Figure 8 Relationship between PLK4 expression and immune cell infiltration in GBM. The effect of PLK4 expression on immune cell infiltration in GBM patients with high and low PLK4 expression of TCGA **(A)**, CGGA_693**(B)**, and CGGA_325 **(C)** datasets via ImmuCellAl algorithm. **P*<0.05, ***P*<0.01, ****P*<0.001.

Supplementary Figure 9 Correlation analysis between PLK4 and immune and inflammatory-related metagenes in GBM. In TCGA **(A)**, CGGA_693 **(B)**, and CGGA_325 **(C)** datasets, we analyzed the enrichment score of seven immune and inflammatory-related genes. **P*<0.05, ***P*<0.01, ****P*<0.001, *****P*<0.0001.

Supplementary Figure 10 scRNA-seq results for PLK4 in GBM. **(A)** Cells were annotated into seven clusters and were annotated as myeloid cells, neoplastic cells, oligodendrocyte precursor cells, oligodendrocytes, astrocytes, neurons, and vascular cells. **(B)** Violin plot of PLK4 expression distribution of seven cell clusters. **(C)** Cells were annotated into eight clusters and were annotated as neoplastic cells, oligodendrocytes, M2 macrophages, M1 macrophages, vascular cells, oligodendrocyte precursor cells, astrocytes, and neurons. **(D)** Four main branches can be seen in neoplastic cell trajectory analysis. Cells are colored based on states (left), pseudotime (middle), and PLK4 expression (right). **(E)** M1 macrophage trajectory analysis reveals seven branches. Cells are colored based on states (left), pseudotime (middle), and PLK4 expression (right).

Supplementary Figure 11 PLK4 is correlated with cell cycle and proliferation in glioma. The expression levels of the PLK4 mRNA **(A)** and protein **(B)** in human astrocyte (HA) and five different glioma cell lines (U87, LN229, U251MG, A172, and T98). **(C)** The related expression of PLK4 mRNA and protein after knocking down *PLK4* gene via siRNA in U87 and LN229. Cell cycle distribution was evaluated using flow cytometry after knocking down *PLK4* gene via siRNA in U87 **(D, E)** and LN229 **(F, G)**. EdU assays were employed to measure cell viability and proliferation after knocking down *PLK4* gene via siRNA in U87 **(H)** and LN229 **(I)**. CCK8 assays were employed to measure cell viability and proliferation after knocking down *PLK4* gene via siRNA in U87 **(J)** and LN229 **(K)**.

Supplementary Figure 12 The bioluminescent intensity of the glioma-bearing mice in different groups. **P*<0.05, ***P*<0.01, ****P*<0.001, *****P*<0.0001.
