# Supplementary figures and images for: PLK4 initiates crosstalk between cell cycle, cell proliferation and macrophages infiltration in gliomas
Source: Front Oncol. 2022 Dec 22;12:1055371. doi: 10.3389/fonc.2022.1055371 (PMC9815703; doi:10.3389/fonc.2022.1055371)

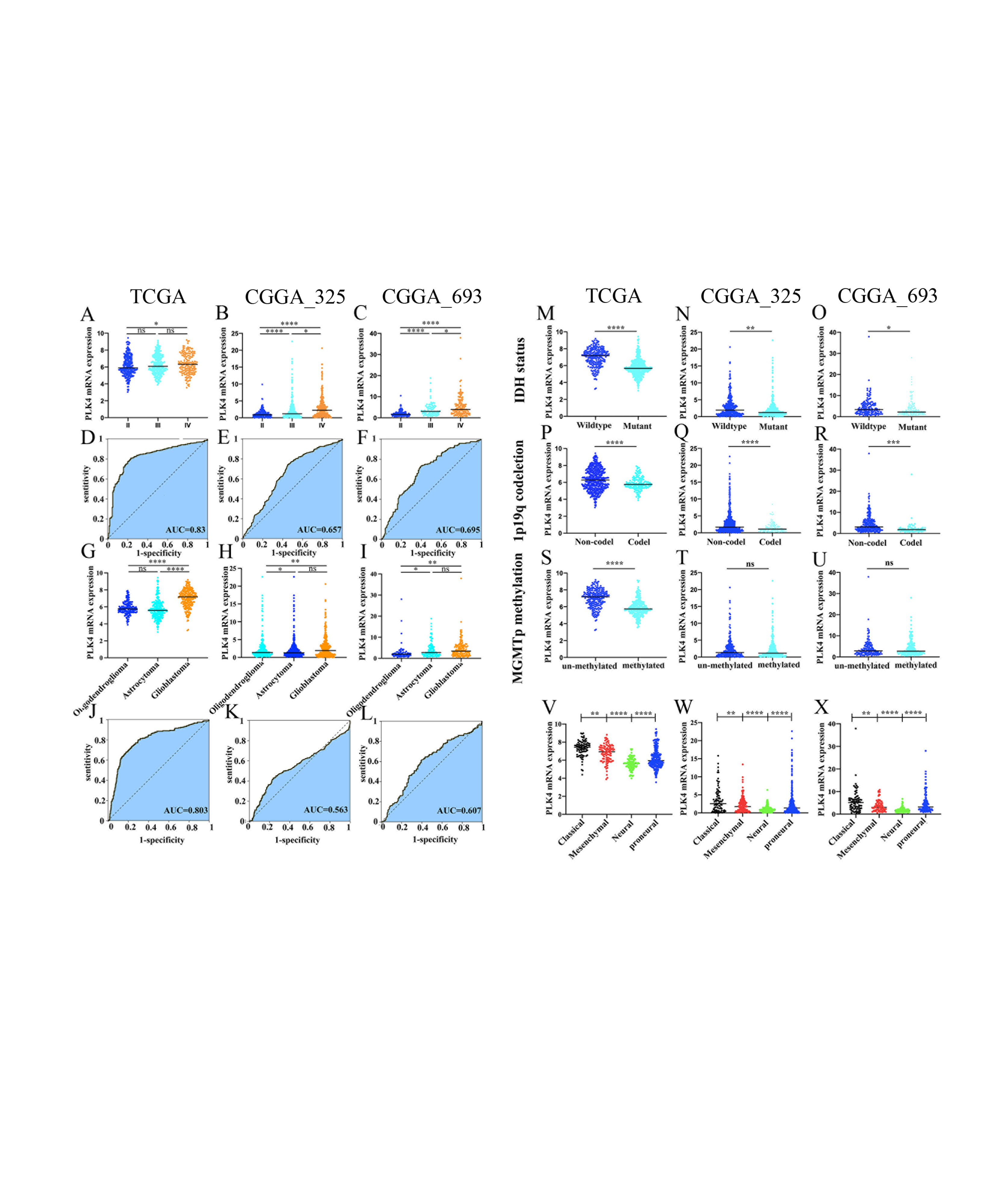

Supplement: Supplementary Figure 1 — PLK4 expression signature in CGGA and TCGA databases. The expression level (A-C) of PLK4 in WHO II, WHO III, and WHO IV glioma and ROC curve (D-F) analysis via TCGA, CGGA_325, and CGGA_693 databases. The expression level (G-I) of PLK4 in oligodendroglioma, astrocytoma, and glioblastoma and ROC curve (J-L) analysis via TCGA, CGGA-325, and CGGA-693 databases. The expression level of PLK4 in different IDH status (M-O), 1p/19q status (P-R) and MGMT promotor status (S-U) via TCGA, CGGA_325, and CGGA_693 databases. The expression level (V-X) of PLK4 in glioma subtypes via TCGA, CGGA-325, and CGGA-693 databases. *P<0.05, **P<0.01, ***P<0.001, ****P<0.0001. [file Image_1.tif]

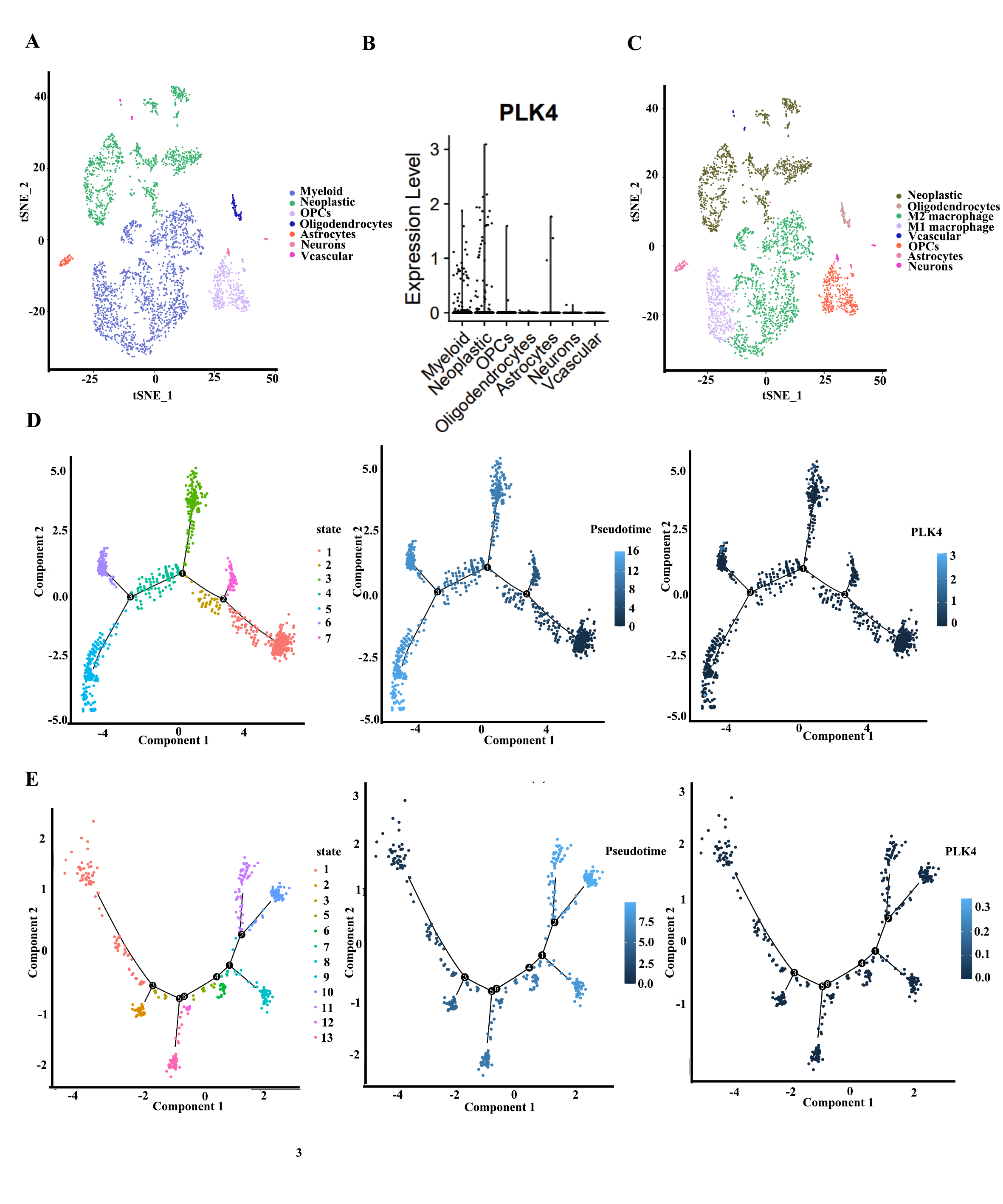

Supplement: Supplementary Figure 10 — scRNA-seq results for PLK4 in GBM. (A) Cells were annotated into seven clusters and were annotated as myeloid cells, neoplastic cells, oligodendrocyte precursor cells, oligodendrocytes, astrocytes, neurons, and vascular cells. (B) Violin plot of PLK4 expression distribution of seven cell clusters. (C) Cells were annotated into eight clusters and were annotated as neoplastic cells, oligodendrocytes, M2 macrophages, M1 macrophages, vascular cells, oligodendrocyte precursor cells, astrocytes, and neurons. (D) Four main branches can be seen in neoplastic cell trajectory analysis. Cells are colored based on states (left), pseudotime (middle), and PLK4 expression (right). (E) M1 macrophage trajectory analysis reveals seven branches. Cells are colored based on states (left), pseudotime (middle), and PLK4 expression (right). [file Image_10.tif]

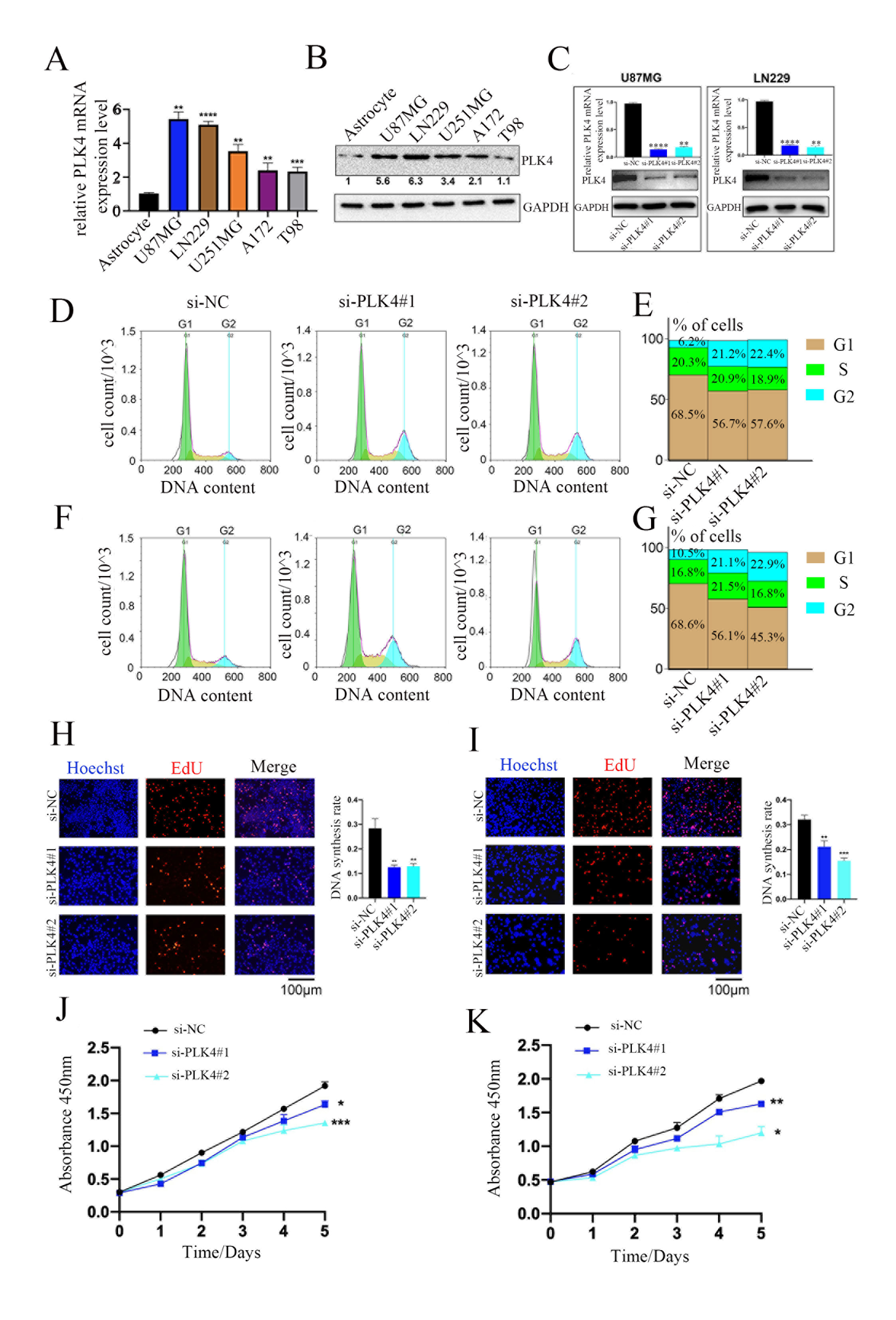

Supplement: Supplementary Figure 11 — PLK4 is correlated with cell cycle and proliferation in glioma. The expression levels of the PLK4 mRNA (A) and protein (B) in human astrocyte (HA) and five different glioma cell lines (U87, LN229, U251MG, A172, and T98). (C) The related expression of PLK4 mRNA and protein after knocking down PLK4 gene via siRNA in U87 and LN229. Cell cycle distribution was evaluated using flow cytometry after knocking down PLK4 gene via siRNA in U87 (D, E) and LN229 (F, G). EdU assays were employed to measure cell viability and proliferation after knocking down PLK4 gene via siRNA in U87 (H) and LN229 (I). CCK8 assays were employed to measure cell viability and proliferation after knocking down PLK4 gene via siRNA in U87 (J) and LN229 (K). [file Image_11.tif]

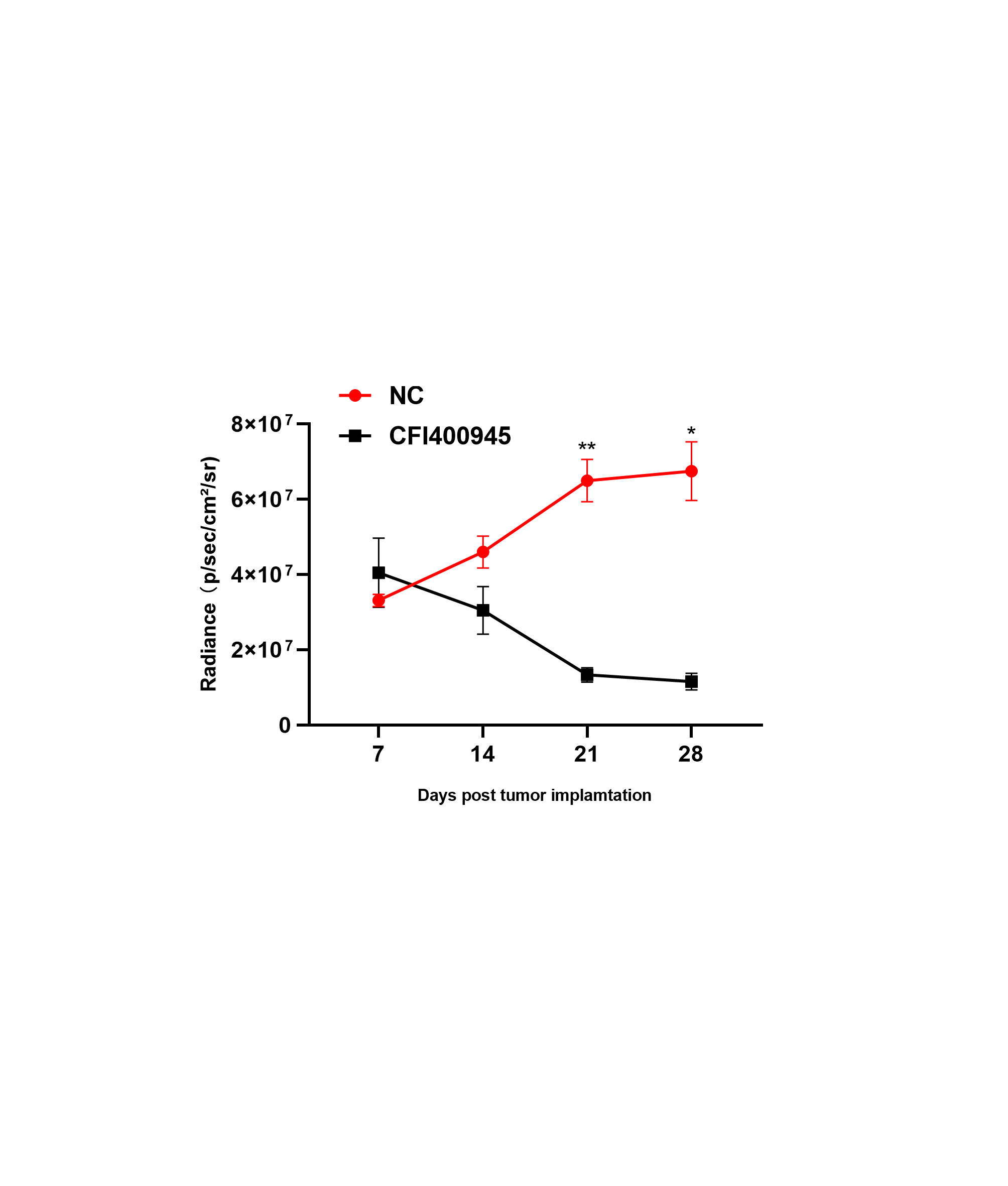

Supplement: Supplementary Figure 12 — The bioluminescent intensity of the glioma-bearing mice in different groups. *P<0.05, **P<0.01, ***P<0.001, ****P<0.0001. [file Image_12.tif]
